# Gallstones; Their Surgical Treatment1Read at the 36th annual meeting of the Medical Association of Central New York, held at Auburn, September 22, 1903.

**Published:** 1904-02

**Authors:** B. Farquhar Curtis

**Affiliations:** New York; Professor of the Principles of Surgery, University and Bellevue Hospital Medical College; Surgeon to Saint Luke’s Hospital, etc.


### BUFFALO MEDICAL JOURNAL.

VOL. XLIII.—LIX. FEBRUARY, 1904. No. 7.

### ORIGINAL COMMUNICATIONS.

### Gallstones; Their Surgical Treatment.[1]

BY B. FARQUHAR CURTIS, M. D., New York.

Professor of the Principles of Surgery, University and Bellevue Hospital Medical College; Surgeon to Saint Luke's Hospital, etc.

THE last few years have greatly enlarged our knowledge of the pathology of gallstones and altered our views of the proper treatment of the various conditions caused by them. Many of these changes are analogous to those which have taken place in our opinions of appendicitis and its treatment. In both we have conditions which may be treated medically, that is to say, expectantly, for little direct effect can be produced upon either by medical or hygienic measures, and in both the only real treatment for well marked lesions is surgical and operative.

In both conditions, too, the technique of operation has been so far developed that surgeons are agreed as to essentials, and all the various possible conditions and complications have been met and overcome with appropriate operative procedures. In appendicitis practical unanimity prevails as to what treatment is to be adopted in the different types of the disease, those which require operation immediately, those which can wait for a convenient season, and those which may escape without operation. Less unanimity reigns in the case of gallstones, and it is the object of this paper to attempt to formulate some ideas upon the various cases in which operation is necessary, advisable, or can be dispensed with.

It will be best to give a brief recapitulation of the present status of gallstone pathology as a foundation for our arguments. Gallstones are formed by deposits of the biliary constituents around a foreign body, usually a clump of mucus produced by

1. Read at the 36th annual meeting of the Medical Association of Central New York, held at Auburn, September 22, 1903.

slight catarrhal inflammation of the gallbladder. This inflammation is due to infection which reaches the gallbladder by ascending the ducts from the duodenum, by penetrating the walls of the bladder from some neighboring focus (suppuration of the kidney, or abscess around the vermiform appendix, for instance); or, finally, by way of the bloodvessels. The last mode of infection is supposed by Chiari and Councilman to be the most frequent. It is analogous to the cases of osteomyelitis of a bone of the extremities secondary to an abscess of the tonsil.

Stones very rarely form in the gallducts, those which are found there having originated in the bladder. Obstruction to the free outflow of the bile hastens the growth of the calculi. When a stone becomes lodged in the neck of the bladder or in the cystic duct it blocks the outflow of the vesical contents, and the intravesical pressure is increased by secretion of mucus and by inflammatory exudate. There are three modes of termination in such a case:

(a) The stone remains in place, blocking the passage, resulting in (1) permanent distension of the gallbladder with mucus, serum or pus; (2) perforation or gangrene of the gallbladder; or in (3) subsidence of the attack when the bladder walls become accustomed to the pressure and resume their powers of absorption, thus removing a portion of the fluid contents.

(b) The stone becomes dislodged, falling back into the bladder, and all the symptoms are relieved.

(c) The stone passes on into the common duct, with the result that, (1) the stone passes on into the bowel; (2) the stone remains in the common duct, causing chronic biliary obstruction, or not obstructing the duct and causing few or no symptoms (becoming "latent" in the last case); or that (3) perforation of the duct occurs, which may result in escape of the stone into the bowel or elsewhere, by the fistula.

In any position of the stone, and at any time in the course of the case, the so-called "latency" may ensue if the stone does not obstruct the biliary passages, and if there is no active infection of the biliary system. This latency may be terminated at any time by infection setting up inflammation or by traumatism altering the position of the stone.

In gallstone pathology there are two main features, obstruction of the biliary passages at some point, and inflammatory complications due to infection, and although the majority of cases combine the two, it is possible to divide them into two practically distinct classes, according to the presence or absence of infection. Infection is at the beginning of the formation of the calculi, and it is also the principal source of the dangers arising from them.

If a gallbladder containing stones becomes infected, suppuration. ulceration, and perforation with all their disastrous consequences are more likely to take place than in a bladder free from stones. This is true whether the stones are so placed as to obstruct the outlet of the bladder or not, although obstruction increases the danger. The same is true of a stone in the common duct, for infection greatly enhances the dangers of that serious condition.

A mild cholecystitis, then, may cause the formation of gallstones, and the presence of the calculi predisposes to a grave cholecystitis with perforation, peritonitis, and the like. These consequences of cholecystitis should be borne in mind by the practitioner, who is rather inclined to underrate the importance of this inflammation, and in fact often overlooks its existence, for it is much more common than is generally supposed. When he meets an attack of colicky pain in the upper part of the abdomen, without jaundice, and without a previous history of attacks of biliary colic, he thinks the trouble cannot be in the biliary system and he talks vaguely of "taking cold," or of gastric or intestinal colic. But the reasoning is incorrect, for a cholecystitis may exist before gallstones are formed, and jaundice is not a usual symptom of cholecystitis. Not infrequently cholecystitis is brought to the surgeon with the diagnosis of appendicitis, and my first case of gallstones was operated upon with that diagnosis. The gallbladder lay low down in the iliac fossa, and the unusually low area of liver dulness was supposed to be caused by a collection of feces in the transverse colon. The recognition of cholecystitis, however, in most cases is easy, for the pain and tenderness correspond to the position of the gallbladder, and the latter can often be distinctly felt. Every case of cholecystitis demands energetic treatment whether gallstones are present or not, and if the symptoms are violent or do not yield soon to treatment, surgical aid should be invoked.

Few will disagree with the general statement that all cases of gallstones complicated with sepsis require surgical treatment. The seriousness of these conditions is illustrated by the following cases:

Charles N., 29 years of age, admitted to Saint Luke's Hospital, October 28, 1900, had general peritonitis of obscure origin. An immediate operation was performed, incision being made over the vermiform appendix which was found normal. A median incision higher up with transverse cut across the right rectus showed advanced cholecystitis. The gallbladder was isolated, aspirated, and secured in the wound with a drain in it. The abdomen was full of sero-pus and was also drained. The patient rallied but died of sepsis forty-eight hours later.

Christine S., 67 years of age, who had been ill for some days with an acute cholecystitis, was admitted to Saint Luke's Hospital, January 18, 1900, in a threatening septic condition, with high fever and rapid pulse. An incision was made over the gall-bladder which could be felt plainly, and revealed that organ distended with mucus, and its walls sloughing. It was opened, six small stones removed, and a drain secured in it. The patient did not improve, and died in forty-eight hours with the signs of a septic bronchitis or bronchopneumonia.

Vincent D., 65 years of age, was transferred to my service from the medical side at Saint Luke's Hospital. He stated that one year before he had had an acute attack of pain over the abdomen, growing worse at night and lasting to the present time, with vomiting at intervals, but he never vomited blood. He had lost flesh and strength. He had a dark greenish jaundice, the mucous membranes were very pale. No changes could be detected in the liver or gallbladder. He coughed continually and subcrepitant râles existed on both side of the chest. Patient had a high septic fever with chills and was in a miserable condition. He had extensive endarteritis. An exploratory laparatomy done December 1, 1900, showed no great external changes in the gall-bladder or liver. The bladder was distended with bile, but not tense, and without marked inflammatory changes in the walls. No calculi were found. Drainage of the gallbladder was established. Cultures from the bile revealed growths of streptococci and staphylococci. The patient died thirteen days later of continued sepsis. Autopsy showed simply inflammation of the bladder and ducts without abscess formation.

A latent case of great interest is the following:

Edward S. B., 53 years of age, was admitted to Saint Luke's Hospital, November 1, 1896. He had never had any symptoms referable to the biliary system. About one month before he had a sudden attack of fever with chills, and pain in the sciatic nerve, lasting two weeks. An abscess then formed in the back, another in the axilla, and the right testicle became swollen. The axillary abscess was drained after entering hospital, the other was already draining, but the sepsis was far advanced and the patient died four days later. The autopsy showed as the only possible source of the general sepsis a cholangitis and cholecystitis. The gall-bladder was adherent, distended with thin pus, and contained a large number of gallstones. In the kidneys were a number of small foci of pus in the cortex and pyramids. There was a large pus cavity in the right testicle. The abscesses in the chest had no direct connection with the gallbladder and must have originated from embolic infection.

Next in gravity to the septic cases are those of continuous, complete, or nearly complete, obstruction of the common duct without infection. These cases are also generally referred to the surgeon, although even yet surgical aid is frequently not sought until the patient's condition is so impaired that even with the most skilful treatment death follows from cholemic failure of the heart, or uncontrollable hemorrhagic oozing. Good results from operative measures in such cases can only be expected when the patient is still in good condition. Even without the shock of operation, fatal consequences may ensue. Cholemia is dangerous, and so is the exhaustion of the patient from suffering, fever, and limited diet, while long-continued obstruction will hasten the progress of the hepatic cirrhosis and interstitial nephritis which are such common accompaniments of cholelithiasis. In one case of stone in the common duct operated upon by me in private, a successful convalescence from operation (choledochostomy, with removal of a single stone) was followed by only temporary relief, and death ensued about five months later from extensive cirrhosis of the liver and interstitial nephritis. The patient, a woman 50 years of age, had endured almost complete obstruction of the duct for some months and although both liver and kidney disease must have antedated the beginning of the jaundice and were doubtless too far advanced even then for a complete recovery, there can be no question that the degenerative changes were greatly hastened by the biliary obstruction.

Turning now to the simple cases of biliary colic or cholecystitis due to gallstones in the bladder or in the cystic duct, without septic complications, it must be granted that the great majority of these patients pass safely through the attack without surgical assistance. But the same questions often arise here as in appendicitis—namely, shall we operate during the attack? shall we operate after the attack has passed?

During the attack the immediate danger of the patient is decidedly less than in acute appendicitis. The severity of the symptoms furnishes the grounds for the decision as to the necessity for operation. The usual symptoms are pain in the epigastrium, sometimes radiating, more or less febrile disturbance, with or without leucocytosis, nausea, vomiting, abdominal tenderness, most marked over the gallbladder (which may be displaced), and rigidity of the right rectus muscle. The distended gallbladder often can be felt. It used to be thought that jaundice never occurred in these simple cases, and even such a good authority as Robson (On gallstones, London, 1892, p. 81) has stated that it is an indication of cancer. More recent experience has demonstrated that jaundice is tolerably common. Riedel holds (Pathogenese, Diagnose, und Behandlung des Gallensteinleidens,

Jena, 1903, S. 14) that this jaundice is due to infection traveling to the ducts from the infected contents of the inflamed gallbladder, claiming that he has always found clearly marked evidence of such infection in the softened and altered character of the liver tissue observed in the frequent opportunities afforded in his operations upon such cases. The general view, however, is that the jaundice is due to a mechanical cause, the pressure of the stone lying in the cystic duct or neck of the bladder, or the pressure of the distended gallbladder itself. The distended gallbladder can obstruct the common duct by direct pressure, or by traction, especially when adhesions exist as they commonly do. The truth is that probably both views are correct. The following cases can be related in favor of the mechanical causation of jaundice in nonseptic conditions:

Margaret M., a patient of Dr. L. A. Coffin, 20 years of age, was operated upon by me at her house for suppurative appendicitis March 31, 1900. She had well-marked jaundice at the time, and stated that she had had frequent previous attacks of jaundice, but never any symptoms of appendicitis before this attack. The attacks of jaundice had the ordinary character of catarrhal jaundice. She had a brother about the same age who was also liable to attacks of jaundice and, further, her mother had been liable to jaundice all her life and had died of some liver affection when 30 years old. The appendix was removed at the operation, the abscess cavity drained, and the symptoms referable to the appendix gradually ceased. The temperature fell to normal, but the pulse continued rapid and feeble. Jaundice and vomiting continued for a week. The wound was healing well, but the patient gradually failed and died twelve days after the operation. Autopsy showed the liver and spleen greatly enlarged. The gallbladder, much distended, was obstructed by a single stone at the entrance of the cystic duct. The cystic duct made a sharp *s*-curve and the protruding neck of the distended gallbladder lay in contact with and compressed the common duct, and also pressed distinctly upon the pylorus. The abscess cavity was filled up and the operation wound granulating. There was no peritonitis and no cirrhosis of the liver. If we had known these facts we would have operated upon the gallbladder, but the patient was too feeble for an extensive operation and the frequent recovery from similar attacks and the fact that these had always seemed to be ordinary catarrhal jaundice are sufficient excuse.

A woman 54 years of age, with chronic jaundice, was operated upon November 9, 1897, at Saint Luke's Hospital. I did a cystostomy, finding a single large stone in a contracted gallbladder pressing upon the choledochus, and removed the calculus. Bile flowed through the drainage sinus for three weeks, then the patient left the hospital cured.

In cases of gallstone in the gallbladder or cystic duct none of the symptoms enumerated above precludes a safe recovery by the passage or by the disengagement of the stone, or by relief of tension when absorption of the contents of the gallbladder takes place. The ordinary measures of palliative and expectant treatment are allowable up to a certain point. But in my judgment if the symptoms persist unaltered for a long time (two or three days in the severe cases, a week in the milder ones) there is serious danger of gangrene or perforation of the bladder, peritonitis, or other grave septic complication.

The special signs of danger of septic accidents are, increasing rigidity of the abdominal muscles, and an increasing leucocytosis, blood examinations being made at intervals of a few hours. A rising temperature is less threatening and the pulse is valueless as an indication of danger. The same must be said of nausea and vomiting, for they are so common in this condition that they lose the usefulness as danger-signals which they possess in appendicitis. In all cases with undue persistence of ordinary symptoms, or with the development of such signals of danger, operation should be undertaken if possible before serious complications have developed. The risks of delay in suspicious cases are far greater than those of operation.

When the acute attack is over, the patient may be entirely free from symptoms, whether the stone has passed, or been dislodged, or the bladder has been relieved by absorption of its contents, and often it is impossible to say which of these modes of recovery has prevailed in the particular case. But even when the stone has been proved to have passed, by being recovered in the feces, there is a probability that others remain which may give occasion to trouble later. According to Riedel (loc. cit., p. 2) in 44 per cent. of his cases in which gallstone colic attacks had occurred there was a calculus in the bladder too large to pass the cystic duct, the attacks having been abortive; therefore we may assume that nearly one half of the patients still retain the offending calculus in the gallbladder after an attack of biliary colic. Mayo states (Boston *Med. and Surg. Jour.*, 1903, cxlviii., p. 545), "We have never operated upon a patient who has passed calculi that we have not found more in the gallbladder." I would not, however, be willing to make the extreme statement that all such cases must have stones remaining. In Saint Luke's Hospital, December 12, 1900, I operated upon a gentleman 55 years of age, with chronic obstructive jaundice, which had lasted in variable degrees for a year. A stone in the common duct was considered certain and the diagnosis was confirmed by Dr. E. G. Janeway. The gallbladder was contracted and adherent, but contained no stones. The common duct was opened and explored, no stone found and the passage demonstrated free to the bowel, having a diameter of three-eighths of an inch at the papilla. The duct was drained, the gallbladder freed from adhesions and left in place. Bile flowed only nine days, and the sinus closed in about four weeks. The patient had remained perfectly well, when last seen 2¼ years after the operation. We concluded that the obstructing calculus had passed through the papilla just before the operation.

In another case which was operated upon at my clinic at Bellevue Hospital last spring by a visiting surgeon from Europe, who has had a wide experience in gallstone surgery, after he had agreed in the diagnosis of stone in the common duct, only a few very small stones were found in the bladder and none in the common duct. The duct was found widely dilated and it was considered probable that the stone had passed just before the operation. The patient was a woman 49 years of age, with no previous attacks of jaundice, who had had pain in the region of the gallbladder and jaundice for several weeks. The gallbladder was contracted and surrounded with dense adhesions. All the stones seen were too small to have caused obstruction of the common duct. These cases show that a single stone may cause obstruction for a long time, and yet may not be so large but that it may be passed—and passed on the eve of operation.

In the great majority of cases, however, calculi will be found after the attack has passed off, even if the originally obstructing stone has reached the bowel safely. A careful study of such patients will often disclose slight and rather indefinite symptoms, such as various forms of "indigestion" or dyspepsia, requiring them to be very careful in their diet or even reducing them to actual invalidism, just as one finds various slight ailments in patients with chronic appendicitis which disappear after removal of the appendix. I have usually found that removal of gallstones which had given no symptoms improved the health or at least the sense of well-being of the patient. They often exclaim with joy, "I find that I can eat everything now." After the attack has been recovered from, a careful examination of the patient may also reveal signs of remaining stones or an inflamed gallbladder such as continued resistance in the rectus muscle, or a little tenderness over the bladder, or possibly the calculi can actually be felt against the vertebral column (Riedel). If symptoms persist, or even when there are no symptoms in the interval, but frequent recurrent attacks take place it may be considered certain that calculi or some serious lesion of the gallbladder exist which can be definitely cured by operation.

The advantages of operation performed while the stones still remain in the gallbladder or cystic duct and before grave infection has developed are numerous.

1. The serious accidents of infection are avoided and any inflammatory condition which may exist can be improved or cured just as drainage cures urinary cystitis.

2. The stone is removed before it enters the common duct, thereby preventing all the dangerous consequences likely to follow the presence of a stone in that passage.

3. Further attacks of cystitis and colic are prevented.

4. Further calculus formation is prevented or impeded, for the stones are formed in infected gallbladders and the latter may be removed by operation or rendered so healthy by drainage that no more calculi will form. Kehr (*Münchener med. Wochenschrift,* 1902, Nos. 41, 42 and 43) estimates the recurrences of stone, adhesions, and other complications after operation in his cases at 10 per cent. Schott (*Beiträge zur klin. Chirurgie,* xxxix., 1903, S. 427) gives the final result of 180 cases from Czerny's clinic followed for five or six years after operation, including serious conditions requiring choledochotomy, cholecystenterostomy, and the like, and found only 5 per cent. had symptoms referable to the biliary system. Not a single case had another gallstone form after operation.

5. It must be remembered also that latent cases are by no means free from danger, so that the individual who has recovered from an attack of colic cannot be considered cured even if he is entirely free from symptoms for years, as it is probable that stones remain behind or that some chronic cholecystitis persists. The possibility of secondary pancreatitis must be kept in mind, and also that of cancer of the gallbladder, which is more common than has been supposed. Riedel states that he has observed in his practice over fifty cases of cancer of the gallbladder, and it is to be noted that while the presence of gallstones is universally accepted as the principal cause of this disease, the stones have generally existed without previous symptoms, the first sign of trouble being given by the tumor of the gallbladder itself.

6. Without reference to more important results, it is the general feeling of those with experience in the surgery of cholelithiasis that in the latent cases, while the patients do not present symptoms pointing directly to the biliary system, they are afflicted with various dyspeptic complaints and that the latter can be relieved or permanently cured by operation.

7 E. Forty-first Street.

